# Safety and Efficacy of Bispecific Antibody Treatment in Relapsed/Refractory Multiple Myeloma: A Systematic Review and Meta-Analysis of Proportions from Clinical Trials

**DOI:** 10.3390/cancers17172727

**Published:** 2025-08-22

**Authors:** Sabrina Bakogeorgou, Charalampos Filippatos, Panagiotis Malandrakis, Anastasios Tentolouris, Evangelos Terpos, Maria Gavriatopoulou, Ioannis Ntanasis-Stathopoulos

**Affiliations:** 1 Department of Clinical Therapeutics, School of Medicine, National and Kapodistrian University of Athens, Alexandra General Hospital, 11528 Athens, Greece; sabbak@med.uoa.gr (S.B.);; 2 First Department of Propaedeutic Internal Medicine and Diabetes Center, School of Medicine, National and Kapodistrian University of Athens, Laiko General Hospital, 11527 Athens, Greece

**Keywords:** bispecific, antibodies, myeloma, relapsed, refractory, teclistamab, elranatamab, talquetamab, linvoseltamab

## Abstract

**Background**: Multiple myeloma (MM) is an incurable plasma cell dyscrasia with particularly adverse prognosis in relapsed, multi-drug refractory settings. The management of those patients is challenging as treatment options are limited. In this context, bispecific antibodies (BsAbs) have recently emerged as promising therapeutic agents, and several have gained regulatory approval. To better understand their impact in MM landscape, we performed a systematic review and meta-analysis assessing their efficacy and safety in patients with relapsed/refractory MM (RRMM). **Methods**: A systematic search was conducted in the PubMed, ScienceDirect, Scopus and ClinicalTrials.gov databases for clinical trials investigating BsAbs for RRMM. Pooled estimates in terms of proportions along with 95% confidence intervals were calculated with random-effects models. The present meta-analysis was performed following PRISMA guidelines and was registered in PROSPERO (ID: CRD420251028553). **Results**: Based on data from six clinical trials involving 850 patients, the pooled overall response and complete response or better rates were 69% and 42%, respectively, whereas the pooled rate of duration of response for at least one year was 71%. The estimated one-year progression-free survival and overall survival were 56% and 72%, respectively. Neutropenia was the most frequently observed severe hematological toxicity, with a pooled incidence of 46%. Grade ≥3 infections occurred in 29%, while any-grade CRS occurred in 69%, as per pooled analysis. Finally, an exploratory minimal residual disease (MRD) analysis in four of the six studies yielded a pooled MRD-negativity rate of 24%. **Conclusions**: BsAbs demonstrated commendable efficacy in heavily pretreated RRMM patients, in terms of response rates and survival outcomes. However, notable rates of hematologic toxicity, infections, and CRS were recorded. These findings support the clinical utility of BsAbs in RRMM, while highlighting the need for comprehensive toxicity management.

## Simple Summary

Multiple myeloma (MM) is an incurable type of blood cancer with dismal prognosis in heavily pretreated, relapsed patients. Recently, novel agents called bispecific antibodies (BsAbs) have shown promising outcomes and several have gained regulatory approval. This article systematically reviewed and meta-analyzed data from six clinical trials on BsAbs for relapsed/refractory MM involving 850 patients (PROSPERO ID: CRD420251028553). The pooled overall and complete response or better rates were 69% and 42%, respectively, whereas the pooled rate of duration of response for at least one year was 71%. The estimated one-year progression-free and overall survival were 56% and 72%, respectively. Neutropenia was the most common severe hematological toxicity, with a pooled incidence of 46%. Grade ≥3 infections occurred in 29%, while any-grade CRS occurred in 69%. Finally, in four of the six studies, the pooled MRD-negativity rate at any point during treatment was 24%. These findings suggest that BsAbs can induce strong and durable responses in these populations, with beneficial survival outcomes. However, they also seem to have a unique safety profile bearing an elevated risk of treatment-related toxicities.

## 1. Introduction

Multiple myeloma (MM) is a malignant plasma cell disorder, characterized by the clonal proliferation of malignant plasma cells within the bone marrow, leading to an overexpression of monoclonal immunoglobulin. In turn, this may lead to end-organ damage including hypercalcemia, renal impairment, anemia and osteolytic disease, as well as myeloma-defining events (increased percentage ≥60% of bone marrow plasma cell infiltration, increased free light chain ratio ≥100, focal lesions on magnetic resonance imaging) that require anti-myeloma treatment [1]. With a growing prevalence over the past 40 years, it represents the second most common hematological malignancy worldwide, accounting for approximately 1–2% of all cancers and 10% of hematologic neoplasms [2]. In 2022, according to Globocan, a total of 187952 new MM cases were identified (39.3% in Asia, 26.7% in Europe, and 19.7% in Northern America), while a total of 121388 patients succumbed to the disease [3]. Despite advances in therapy that have led to significant survival benefits, MM remains incurable for most patients, with the majority eventually experiencing disease progression and becoming refractory to available treatments [4].

Relapsed and refractory MM (RRMM) is associated with poor prognosis, especially in patients who are refractory to multiple drugs. Real-world studies report that median overall survival (OS)—defined as the time from initiation of the respective salvage therapy to death from any cause—for triple-class refractory (disease refractory to a PI, IMiD, and anti-CD38 monoclonal antibody) patients is less than 14 months, and for penta-class refractory patients(disease refractory to two PIs, IMiDs and an anti-CD38 monoclonal antibody) as short as 6 months, even with novel agents [5,6]. Studies have also shown that patients who become refractory to CD38-targeted monoclonal antibodies have a median overall survival of only 8–11 months, and face extremely limited therapeutic options and poor outcomes, increasing the need for innovative therapies [7,8]. Therefore, the clinical management of RRMM patients is challenging, especially in heavily pretreated populations.

Over the past decade, the therapeutic landscape of RRMM has evolved significantly with the introduction of novel agents, including proteasome inhibitors, immunomodulatory drugs, and monoclonal antibodies. Among these, antibody-based regimens have been established as key therapeutic options, leading to significantly improved patient outcomes [9,10]. Specifically, monoclonal antibodies targeting Cluster of Differentiation 38 (CD38), B-cell maturation antigen (BCMA) and Signaling Lymphocytic Activation Molecule Family member 7 (SLAMF7) have demonstrated significant efficacy in RRMM and are now widely used in clinical practice [11].

Recently, bispecific antibodies (BsAbs) have emerged as a promising class of immunotherapeutic agents for RRMM. These molecules are engineered to simultaneously bind to a tumor-associated antigen [such as BCMA or G protein-coupled receptor, class C group 5 member D (GPRC5D)] on myeloma cells and CD3 on T cells, thereby redirecting T-cell cytotoxicity against malignant plasma cells [12,13]. BsAbs have shown deep and durable responses in heavily pretreated RRMM populations within clinical trials, including those with triple-class refractory disease [13]. Therefore, several BsAbs such as teclistamab, talquetamab, elranatamab, and linvoseltamab have received regulatory approvals for the treatment of RRMM patients who have received at least four or five prior lines of therapy, reflecting their clinical impact in this difficult-to-treat population [14,15,16]. The approval of these novel agents has marked a new era in the therapeutic landscape of MM and has generated significant interest, as they seem to offer a promising new treatment option for RRMM patients without many alternatives [17]. In an era where patients could become increasingly exposed to available regimens even earlier in their disease course [18,19], the addition of novel potent therapeutic options in the management of MM is important.

However, questions remain regarding their comparative efficacy and safety, especially as more agents and combinations become available. In this context, we conducted a systematic review and meta-analysis of peer-reviewed and published clinical trials in order to assess the efficacy and safety of BsAbs in RRMM and inform clinical practice.

## 2. Materials and Methods

The present meta-analysis was performed following the Preferred Reporting Items for Systematic Reviews and Meta-Analysis (PRISMA) guidelines [20]. The study protocol was discussed and agreed upon in advance by all authors and was registered in PROSPERO (ID: CRD420251028553).

A systematic search was conducted in the PubMed, ScienceDirect, Cochrane databases and in the ClinicalTrials.gov registry, from inception until March 30, 2025, to identify peer-reviewed and full-text articles of clinical trials on bispecific antibodies for RRMM. The search algorithm used implemented key terms, such as bispecific antibody, BsAb, teclistamab, talquetamab, linvoseltamab, elranatamab, refractory/relapsed multiple myeloma, cevostamab, plasma cell dyscrasia, and was ensured to adhere to the databases’ unique characteristics. The full search strategy is included in the Supplementary Material. Additionally, a comprehensive and systematic snowball approach was used to capture all relevant records by reviewing the reference lists of included studies, ensuring comprehensive coverage and minimizing the risk of omitting previously cited literature [21].

Eligible studies included strictly clinical trials (randomized or not) focusing on adults diagnosed with RRMM who were treated with bispecific antibodies and reported efficacy and safety outcomes in peer-reviewed publications. Exclusion criteria encompassed prospective and retrospective studies, case reports, case series, reviews, in vitro and animal studies, and records not available in English. The screening was conducted independently by two authors, and the final selection was made following consensus. In cases of unresolved disagreement, a third author was consulted (no specific standardized form or software was used).

### 2.1. Data Extraction and Effect Estimates

The data extraction encompassed the following: general information (first author’s name, publication year, database ID), study characteristics (study ID, design, cohort size, follow-up, geographic region), population characteristics (number of males, age), intervention characteristics (BsAb target, drug name), efficacy outcomes [overall response rate (ORR), very good partial response or better rate (≥VGPR), complete response or better rate (≥CR), 1-year duration of response (DoR), 1-year progression-free and overall survival (OS and PFS), and minimal residual disease negativity (MRD-)], and key safety outcomes of hematological or BsAb interest [grade ≥3 anemia, neutropenia, leukopenia, lymphopenia and thrombocytopenia, and any grade cytokine release syndrome (CRS)]. Extracted effect estimates included crude number and percentages for the outcomes alongside their 95% confidence intervals (CI). In studies with multiple intervention arms, the arm receiving the recommended phase 2 dose (RP2D) or the optimal target dose of the investigational drug, as defined by the study authors or regulatory guidelines, was selected for analysis. This approach was used to reflect the dose most likely to be adopted in clinical practice. In case the aforementioned data were not available in the main text, the Supplementary Material was thoroughly screened in order to extract or reproduce the corresponding omitted results. Two authors independently extracted the data, which were then jointly reviewed. Any discrepancies were resolved through consultation with a third author (no specific standardized form or software was used). The finalized data form was reached after team consensus.

### 2.2. Statistical Analysis

Extracted data for continuous numerical variables such as age and follow-up were reported as medians and were extracted as such. An overall analysis of proportions of response rates (ORR, ≥VGPR, ≥CR) and DoR, OS, and PFS at the 1-year timepoint was chosen as the base-case analysis to evaluate the effect of BsAb treatment in RRMM. When proportions of DoR, OS, or PFS at the 1-year timepoints were unavailable, these were either extracted from published Kaplan–Meier survival curves with the tool “WebPlotDigitizer” or calculated directly from patient outcome data provided in the eligible records.

Statistical analysis included pooling of studies and subsequent exploratory sub-analyses. Random-effects models were used to calculate the pooled effect estimates (proportions). Study heterogeneity was assessed by Q-test and I^2^ estimations. Post hoc meta-regression analyses were not able to be performed, as potential variables to be included that introduced heterogeneity had less than 10 entries. Across this analysis, I^2^ < 40% or p (Q-test) < 0.10 was considered low heterogeneity and statistical significance was achieved by *p*-values < 0.05 [22]. All statistical analysis were performed using R/R-Studio version 2024.04.2+764) (Posit Software, PBC, Boston, MA, USA).

### 2.3. Assessment of Study Quality, Risk of Bias and Certainty

All records included strictly peer-reviewed and published articles of registered, non-randomized clinical trials. Risk of bias was assessed by two independent authors with the implementation of ROBINS-I (confounding, selection of participants, classification of interventions, deviations from intended interventions, missing data, measurement of outcomes, and selection of reported results) to our analysis tools [23]. A publication bias assessment was decided not to be conducted in this study, as in the case of meta-analyses of proportions it has been associated with misleading results and its use is not recommended [24,25]. The certainty of evidence for each outcome was assessed using the GRADE approach, starting at low for single-arm studies and downgrading further if appropriate [26].

## 3. Results

### 3.1. Overview of Screening and Included Studies

**Figure 1:**
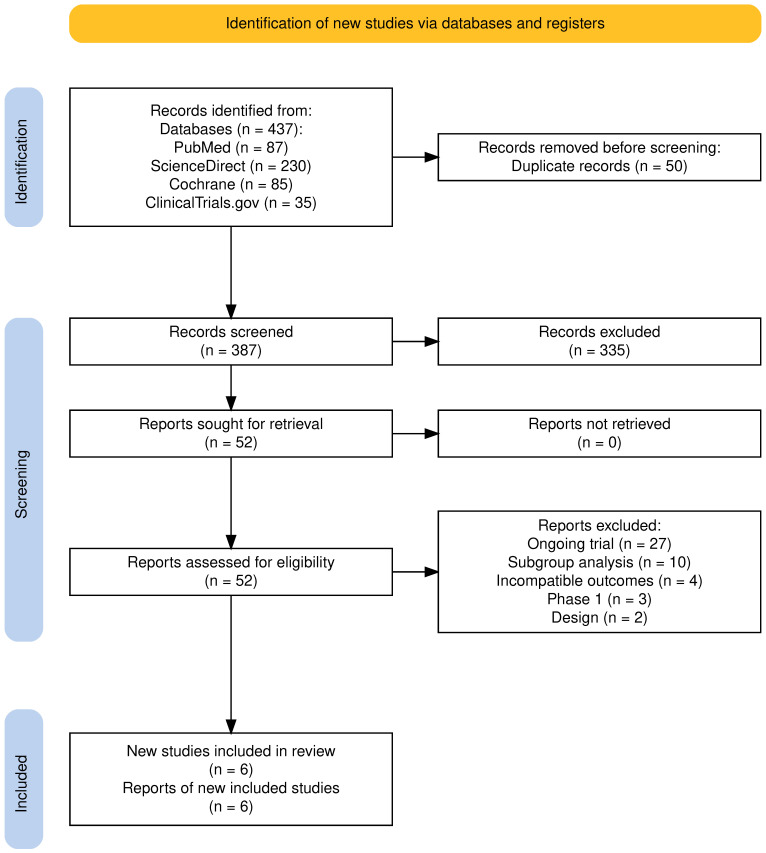
PRISMA 2020 flowchart, step-by-step screening process and selection of studies.

Through the search strategy, 387 records were identified after duplicates were removed. Out of these, the 335 were deemed as irrelevant based on initial screening, resulting ultimately in 52 records assessed for eligibility. After further screening, six records involving 850 RRMM patients from clinical trials on BsAbs met the full eligibility criteria and were included in this analysis [27,28,29,30,31,32]. The following flowchart, created with the PRISMA 2020 Flow Diagram tool, portrays the successive steps in the selection of eligible studies (Figure 1).

All records focused on heavily pretreated patients, with a median of four or more prior lines of therapy. The four out of six included studies investigating BCMA×CD3 targeting agents (teclistamab, elranatamab, linvoseltamab), while two included the GPRC5D×CD3 antibody talquetamab, either alone or in combination with teclistamab. Table 1 presents an overview of the included studies.

### 3.2. Efficacy

#### 3.2.1. Response Rates and Duration of Response Analysis

**Figure 2:**
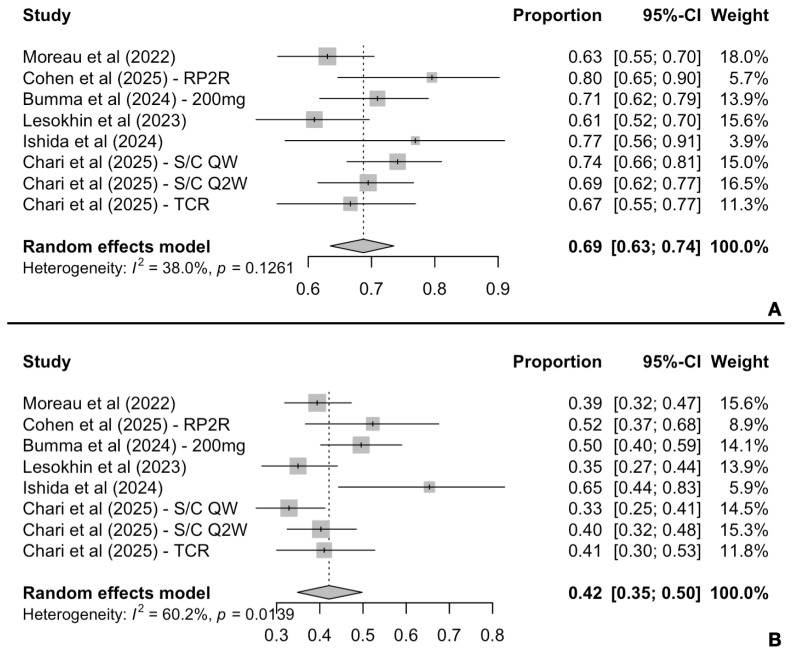
Pooled ORR (**A**) and CR (**Β**) rates by random-effects model.

All six included records reported results for response rates among patients enrolled. The pooled ORR was 69% (95% CI: 63–74%), while heterogeneity was relatively low [I^2^ = 38.0%, P(Q-test) = 0.1261] (Figure 2A). Furthermore, the pooled ≥CR rate was 42% (95% CI: 35–50%) with high variability observed among the included studies [I^2^ = 60.2%, P(Q-test) = 0.014] (Figure 2B).

**Figure 3:**
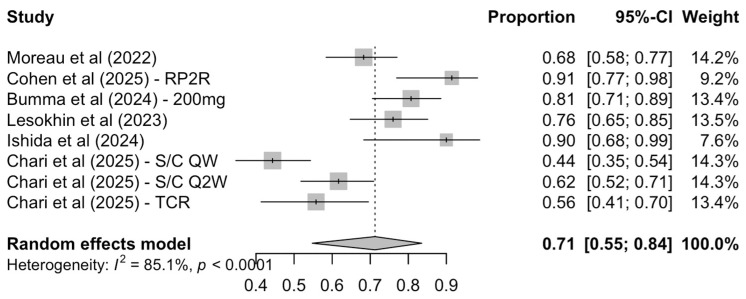
Pooled 1-year DOR rate by random-effects model.

Similarly, all included records reported results with respect to the duration of the achieved response. Specifically, the pooled 1-year DOR rate was 71% (95% CI: 55–84%) while heterogeneity was particularly high [I^2^ = 85.1%, P(Q-test) < 0.001] (Figure 3).

#### 3.2.2. One-Year Survival Rates Analysis

**Figure 4:**
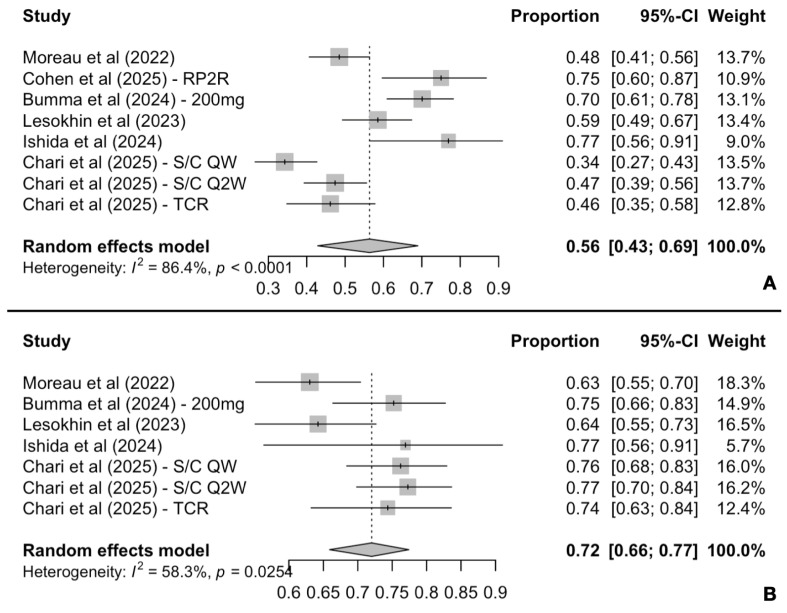
Pooled 1-year PFS (**A**) and OS (**B**) rates.

All six studies reported survival outcomes with data at the 1-year cut-off, both for PFS and OS. The pooled 1-year PFS rate was 56% (95% CI: 43–69%), while heterogeneity was high [I^2^ = 86.4%, P(Q-test) < 0.0001] (Figure 4A). Similarly, the pooled 1-year OS rate was 72% (95% CI: 66–77%), with moderate heterogeneity observed [I^2^ = 58.3%, P(Q-test) = 0.0254] (Figure 4B).

#### 3.2.3. Exploratory Analysis on MRD Negativity Rates

**Figure 5:**
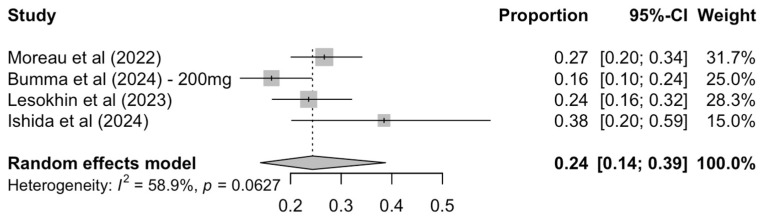
Pooled MRD negativity rate.

MRD negativity rates, assessed at any point during follow-up, were reported in four of the included studies. The pooled MRD negativity rate was 24% (95% CI: 14–39%), with substantial heterogeneity across studies [I^2^ = 58.9%, P(Q-test) = 0.0627] (Figure 5).

### 3.3. Safety

**Figure 6:**
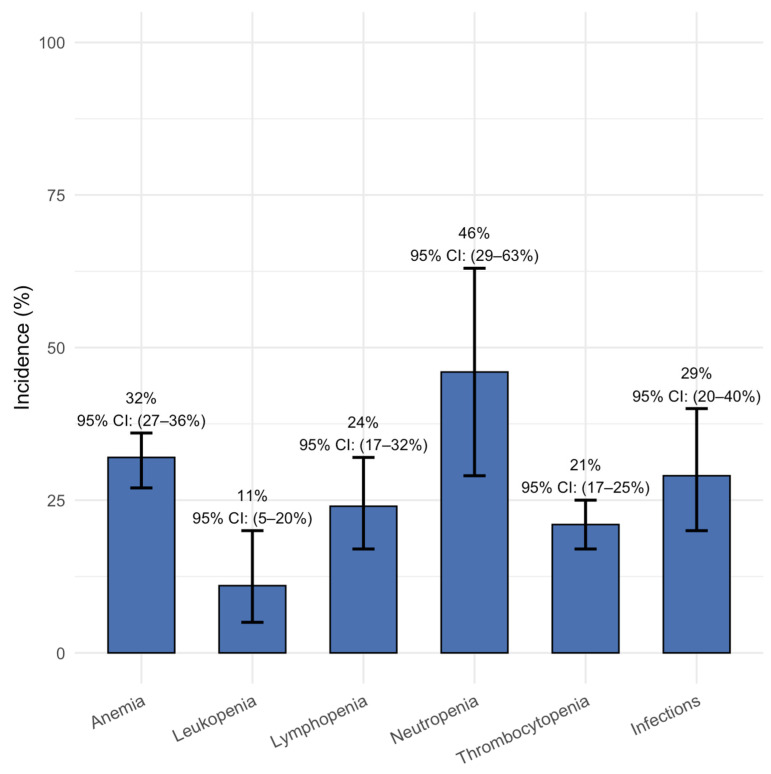
Pooled incidence of grade 3–4 hematologic AEs and infections.

**Figure 7:**
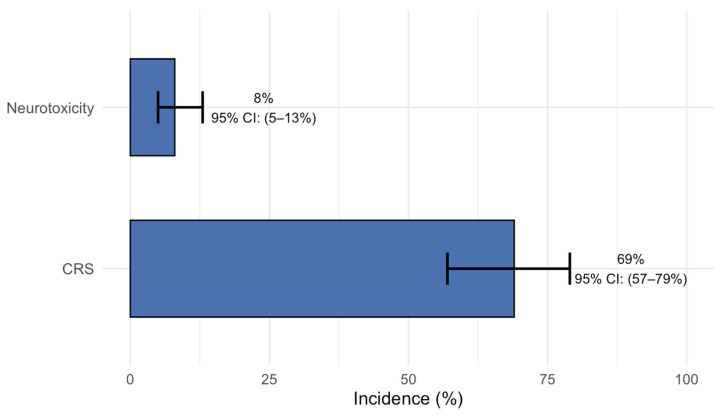
Pooled incidence of any-grade CRS and neurotoxicity.

All included studies reported events of hematological grade 3 or higher adverse events (AEs). Pooled analysis revealed that neutropenia (reduction in neutrophils) was the most frequently observed severe hematological toxicity, occurring in 46% of patients (95% CI: 29–63%). Anemia was observed in 32% (95% CI: 27–36%), followed by lymphopenia (reduction in lymphocytes) in 24% (95% CI: 17–32%), thrombocytopenia in 21% (95% CI: 17–25%), and leukopenia (reduction in circulating leukocytes) in 11% of patients (95% CI: 5–20%). Additionally, grade ≥3 infections were noted in 29% of patients (95% CI: 20–40%) (Figure 6). Any-grade CRS was frequent, with a pooled incidence of 69% (95% CI: 57–79%), while the pooled incidence of any-grade neurotoxic events was 8% (95% CI: 5–13%) (Figure 7).

### 3.4. Risk of Bias and Certainty of Evidence Assessment

Risk of bias was assessed across seven domains adapted from the ROBINS-I tool for single-arm clinical trials. All included studies consistently demonstrated a moderate risk of bias due to confounding, reflecting the inherent limitation of single-arm designs lacking comparator groups and the potential influence of baseline patient characteristics on outcomes. Nevertheless, across all other domains, all studies were deemed as having a low risk of bias, with one exception. Ishida et al. 2024 demonstrated a moderate risk of bias due to missing data. The overall risk of bias was low, supporting the reliability of the included records and of the subsequent pooled outcomes, with cautious interpretation warranted due to the aforementioned limitations (Supplementary Table S1). Specifically, for the MRD negativity rates, there was a high risk of bias due to missing data, as selected patients were evaluated for MRD.

The certainty of evidence for ORR and 1-year OS was rated as low, due to the uncontrolled, open-label nature of all included studies. The certainty for ≥CR rates was also low, with moderate heterogeneity (I^2^ = 60.2%). Outcomes such as 1-year DOR and PFS were graded very low, due both to heterogeneity (I^2^ > 85%) and imprecision. Very low certainty was also assigned to MRD negativity rates, mainly due to missing data. Finally, most safety outcomes were graded as very low due to moderate-to-high heterogeneity.

## 4. Discussion

This meta-analysis provides pooled data from six clinical trials regarding the efficacy and safety of BsAbs targeting BCMA and GPRC5D in patients with RRMM who had received multiple prior therapies. The overall pooled 1-year PFS and OS rates were 56% and 72%, ranging from 34% to 77% and 63% to 77%, respectively. The pooled ≥CR rate was 42% (range 33–65%) and the pooled 1-year DoR was 71% (range 44–91%). The most common grade 3–4 hematologic AEs were neutropenia in 46% (range 21–73%) and anemia in 32% (range 27–36%). Grade 3–4 infections occurred in the 29% (range 20–40%) and were generally manageable.

Overall, our findings indicate that many patients who are treated with BsAbs achieve deep and durable responses, even in advanced disease stages, under a manageable safety profile. Furthermore, they come to further validate the findings of a previous meta-analysis on earlier prospective clinical trials, including those of phase 1, which showed promising efficacy with BsAb treatment [33]. In addition, the integration of GPRC5D-targeting agents, either as monotherapies or in combination with BCMA-targeting agents, seems to offer more favorable treatment responses and tolerability, thus emerging as an important new option for RRMM [34,35,36]. Nevertheless, this was not possible to be evaluated with further statistical rigor in our meta-analysis, due to the limited number of trials included.

Moreover, several of the clinical trials included in this analysis have directly contributed to the regulatory approval of BsAbs in routine clinical practice. These include the pivotal MajesTEC-1 and MonumenTAL-1 trials for teclistamab and talquetamab, respectively, the MagnetisMM-3 trial for elranatamab, as well as the more recent LINKER-MM1 trial for linvoseltamab, which received an accelerated approval in July 2025 [37].

Long-term outcomes from the MajesTEC-1 trial highlight teclistamab’s efficacy and durability, with patients who were treated with a median of fiver prior lines of therapy achieving a notable ORR of 63%, and ≥CR rate of 39%, with a median DoR of 18.4. Grade 3/4 infections, reported in the 55% of the patients were a main safety concern, but overall infection risk seemed to decrease over time [27,38]. Similarly, MonumenTAL-1 showed promising results in all three cohorts of phase 2. These included an ORR of 74% in the QW cohort, 69% in the Q2W cohort, and 67% among those who had received prior T-cell redirection therapies, in RRMM patients with a median of five prior lines of therapy. The study revealed GPRC5D as a new target for bispecific therapy in myeloma, with a safety profile that included lower grade 3–4 infection rates compared to BCMA-targeting agents (20–26%). Despite that, unique GPRC5D-associated toxicities such as nail changes, rash and weight loss were observed, but were usually low-grade and rarely lead to treatment discontinuation [28,39]. Finally, linvoseltamab in the LINKER-MM1 trial represents a promising novel agent in the field, with a commendable ORR of 71% in patients with a median of five prior lines of therapy, making it an effective BCMA bispecific alternative in RRMM [28].

Indeed, BsAbs are transforming the management of RRMM, providing a valuable therapeutic option for heavily pretreated patients, in a time where CAR-T cell therapies are also available. Unlike the latter, which are more complex, time-consuming, and resource-intensive, BsAb off-the-shelf agents allow for rapid treatment initiation and broader accessibility. They can also be administered to a wider patient population; notably, recent studies and meta-analyses suggest that BsAbs achieve commendable response rates, deep remissions, and survival benefits in patients previously treated with CAR-T, anti-BCMA agents or in those with high-risk features [39,40,41,42]. In this context, the final results of the CAMMA 2 study on cevostamab are eagerly awaited [43].

Looking ahead, the future role of BsAbs in RRMM will depend on larger, carefully designed phase 3 trials that include a wider variety of patients and longer follow-up periods. These studies will be crucial to better understand how long these treatments remain effective, how safe they are over time, and their impact on patients’ quality of life. More importantly, they will help physicians figure out the best ways to use BsAbs—whether alone, in combination, or in sequence with other therapies like CAR-T cells or more traditional drugs [44,45]. Future trials should also aim to stratify patients by prior lines of therapy, status of refractoriness, cytogenetic risk, and the presence of comorbidities, to better tailor and personalize treatment decisions [46,47]. Finally, studies with direct comparison between BsAbs and other available therapies will be key to guiding treatment choices and updating clinical guidelines, ensuring patients receive the most effective and personalized care.

The present work synthesizes the most recent and complete data on BsAbs for RRMM, with results strictly from peer-reviewed and published research articles on phase 1/2 or higher clinical trials. This ensures the robustness and reliability of the outcomes analyzed, minimizing the risk of bias by excluding non-peer-reviewed and unpublished sources, such as congress abstracts or early-phase studies with limited interpretability. Furthermore, by focusing solely on clinical trials, a higher level of internal validity, standardized outcome reporting, and more controlled patient populations are ensured. Additionally, by choosing BsAb arms with the recommended drug dosage we aimed to reflect real-world decision-making and regulatory relevance, improving the generalizability and clinical applicability of the findings. Moreover, the use of two independent reviewers for both screening and data extraction, with consensus and adjudication by a third reviewer, strengthens the objectivity and reproducibility of this analysis.

Despite the aforementioned strengths, this work also comes with several limitations. First, the heterogeneity of the included studies, especially in terms of patient characteristics, prior treatments, and dosage, limits the interpretability and applicability of these findings to a wider and more diverse population in real-world settings. Second, due to their uncontrolled and open label nature, all outcomes received a low or very low certainty of evidence. Third, key BsAb-specific side effects are not always explicitly reported, leading to some gaps in our analysis of their unique safety profile. Specifically, for the selected cohorts, GPRC5D-specific AEs such as nail changes, taste changes, and rash were reported only in the record for MonumenTAL-1 and thus a pooled analysis was not feasible. Fourth, the MRD-negativity analysis was only possible in four out of the six included studies and demonstrated substantial heterogeneity [I^2^ = 61.4%, P(Q-test) = 0.0509] and bias due to missingness. Since MRD has increasingly been recognized as a key surrogate endpoint for long-term outcomes in ΜΜ trials [48], the limited availability and variability of MRD data in our analysis represents a limitation to the comprehensive interpretation of treatment efficacy. Additionally, some studies have a median follow-up of just over one year or substantial censoring at 12 months, potentially limiting the reliability of 1-year Kaplan–Meier survival estimates. Finally, the inherent asymmetry of funnel plots and subsequent tests in meta-analyses of proportions does not allow for a statistically sound assessment of publication bias.

## 5. Conclusions

Our meta-analysis, involving data from six clinical trials and 850 RRMM patients, provides robust evidence supporting the efficacy of BsAbs as potent therapeutic options for heavily pretreated patients. These agents yield commendable outcomes and deliver significant benefits in survival and in the depth of responses. These findings underline the impact of this novel drug class on the MM treatment landscape, which can potentially redefine the therapeutic paradigm for RRMM multi-class refractory individuals. Future research should focus on novel BsAbs, combination regimens, optimizing and de-intensifying treatment schedule, toxicity management, and further validating the observed benefits in populations with diverse characteristics and longer follow-up periods.

## Figures and Tables

**Table 1:** Characteristics of the included studies.

Study	Phase	BsAb Target	Drug Name	*n*	FUP (Median)	Age (Median)	Prior LoT (Median)	Triple Class Refractory
MajesTEC-1 Moreau et al., (2022) [24]	I/II	BCMA × CD3	teclistamab	165	14.10	64.00	5.00	128 (77.6)
LINKER-MM1 (200 mg arm) Bumma et al., (2024) [25]	I/II	BCMA × CD3	linvoseltamab	117	14.30	70.00	5.00	96 (82.1)
MagnetisMM-3 Lesokhin et al., (2023) [26]	II	BCMA × CD3	elranatamab	123	14.70	68.00	5.00	119 (96.7)
NCT04696809 Ishida et al., (2024) [27]	I/II	BCMA × CD3	teclistamab	26	14.30	67.50	4.50	17 (65.4)
MonumenTAL-1 (S/C QW arm) Chari et al., (2025) [28]	Ia/Ib	GPRC5D × CD3	talquetamab	143	25.60	67.00	5.00	107 (74.8)
MonumenTAL-1 (S/C Q2W arm) Chari et al., (2025) [28]	Ia/Ib	GPRC5D × CD3	talquetamab	154	19.40	67.00	4.50	110 (71.4)
MonumenTAL-1 (TCR arm) Chari et al., (2025) [28]	Ia/Ib	GPRC5D × CD3	talquetamab	78	16.80	61.00	6.00	66 (84.6)
RedirecTT-1 (RP2R arm) Cohen et al., (2025) [29]	Ib/II	GPRC5D × CD3 + BCMA × CD3	teclistamab + talquetamab	44	18.20	63.00	4.00	37 (84.1)

RP2R: Recommended phase 2 regimen, *n*: number of patients, FUP: follow-up, LoT: lines of therapy, S/C: subcutaneous, QW: once weekly, Q2W: every 2 weeks, TCR: previous T-cell redirection therapy.

## Data Availability

Data available upon request to the corresponding author.

## Supplementary Materials

The following supporting information can be downloaded at: https://www.mdpi.com/article/10.3390/cancers17172727/s1, PICOS, Figure S1: Pooled incidence of grade 3–4 anemia, Figure S2: Pooled incidence of grade 3–4 leukopenia, Figure S3: Pooled incidence of grade 3–4 lymphopenia, Figure S4: Pooled incidence of grade 3–4 neutropenia, Figure S5: Pooled incidence of grade 3–4 thrombocytopenia, Figure S6: Pooled incidence of grade 3–4 infections, Figure S7: Pooled incidence of any-grade CRS, Figure S8: Pooled incidence of any-grade neurotoxic events, Table S1: Risk of bias assessment per domain (adapted ROBINS-I V2), Table S2: Certainty of evidence assessment by GRADE, Table S3: PRISMA 2020 checklist.
