# Supplementary material for: Safety and Efficacy of Bispecific Antibody Treatment in Relapsed/Refractory Multiple Myeloma: A Systematic Review and Meta-Analysis of Proportions from Clinical Trials
Source: Cancers (Basel). 2025 Aug 22;17(17):2727. doi: 10.3390/cancers17172727 (PMC12427194; doi:10.3390/cancers17172727)
Supplement: Supplementary file 1 [file cancers-17-02727-s001.zip › cancers-3830963-supplementary.pdf]

Supplementary Figure S1. Pooled incidence of grade 3-4 anemia

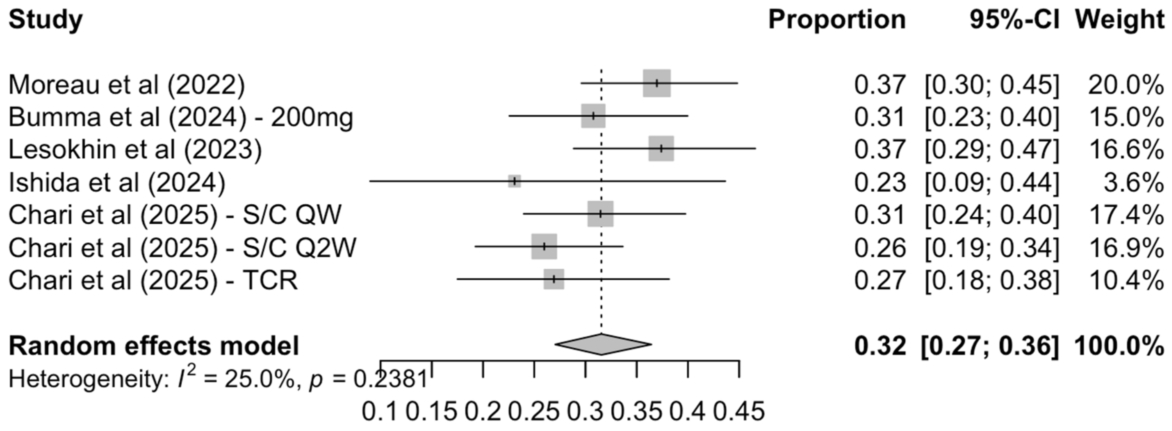

Supplementary Figure S2. Pooled incidence of grade 3-4 leukopenia

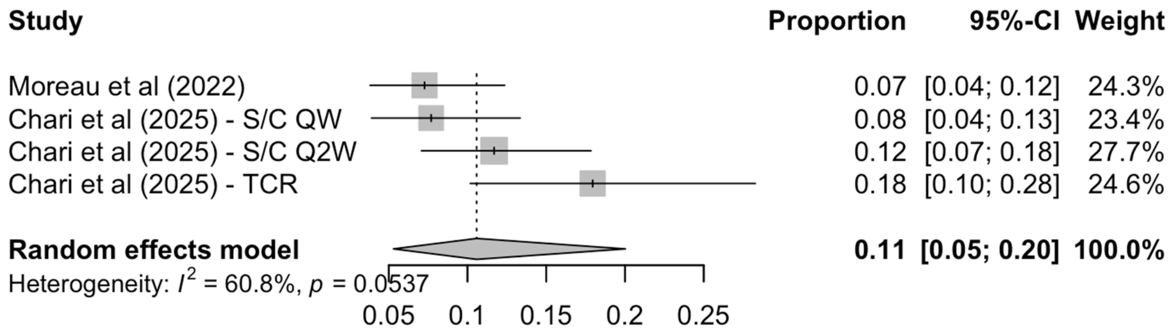

Supplementary Figure S3. Pooled incidence of grade 3-4 lymphopenia

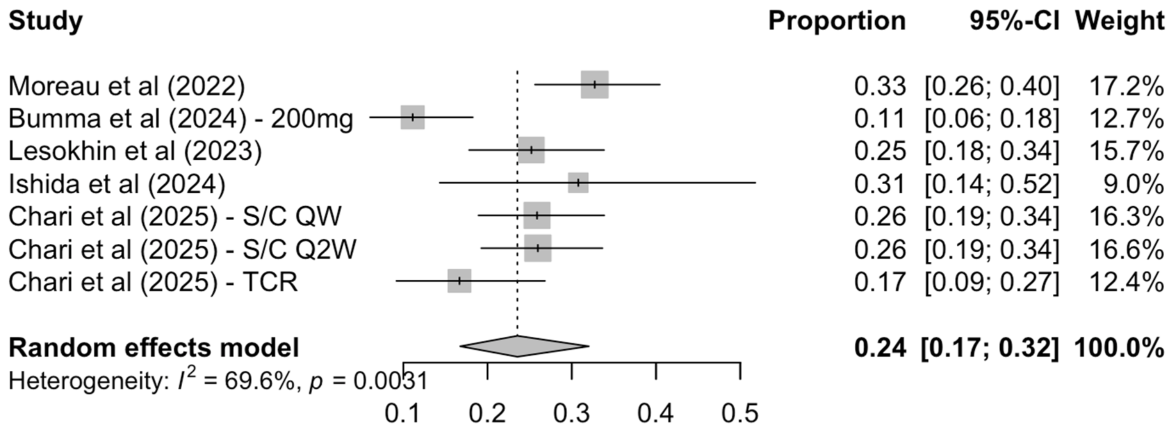

Supplementary Figure S4. Pooled incidence of grade 3-4 neutropenia

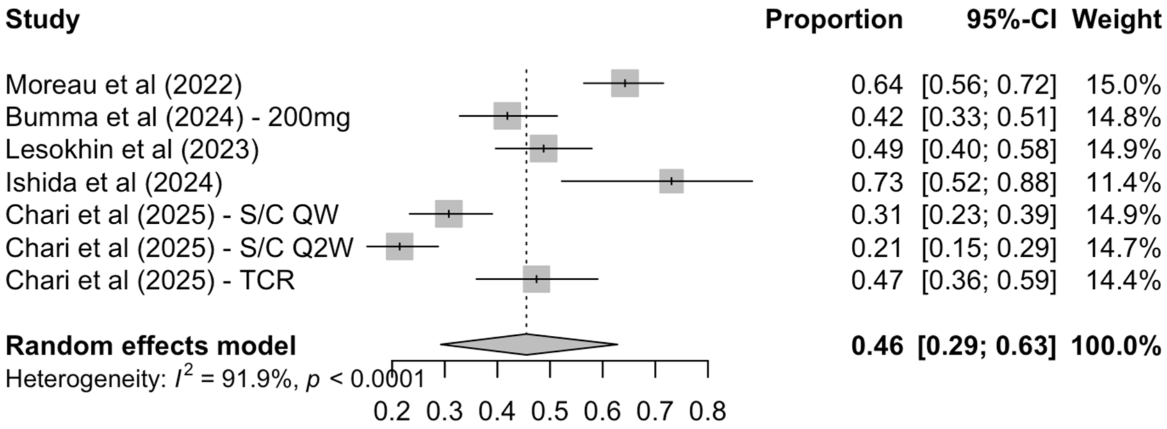

Supplementary Figure S5. Pooled incidence of grade 3-4 thrombocytopenia

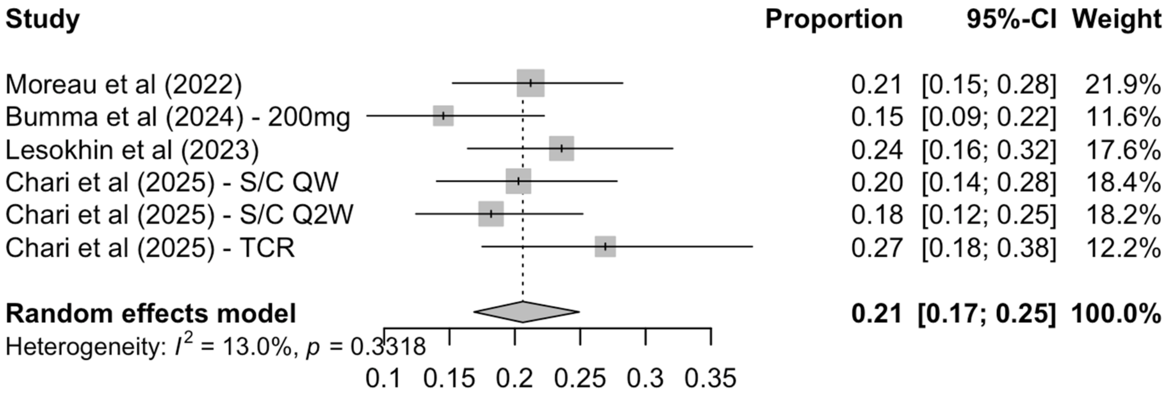

Supplementary Figure S6. Pooled incidence of grade 3-4 infections

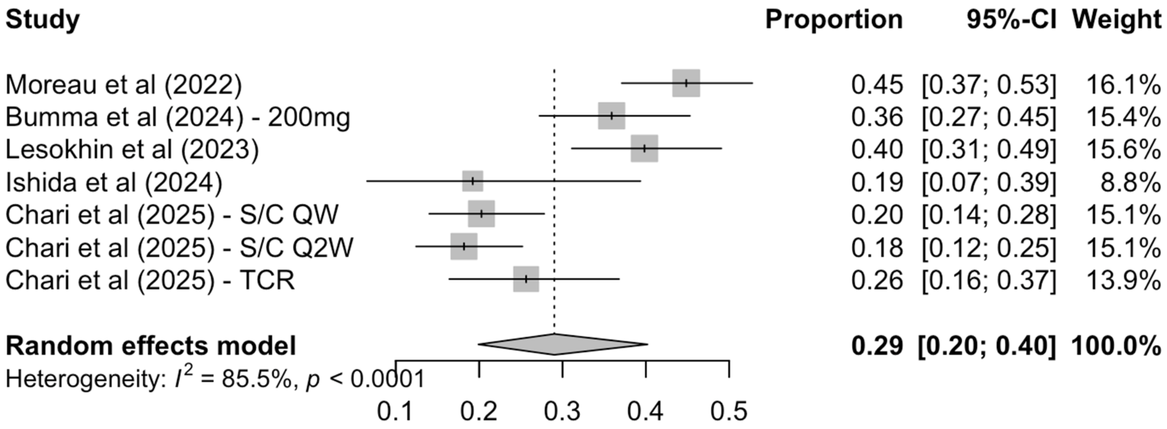

Supplementary Figure S7. Pooled incidence of any-grade CRS

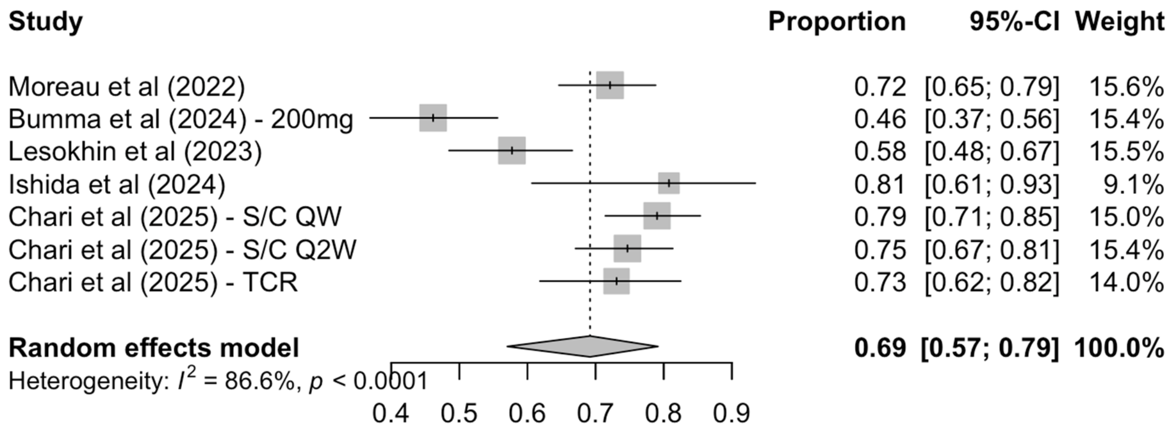

Supplementary Figure S8. Pooled incidence of any-grade Neurotoxicity events

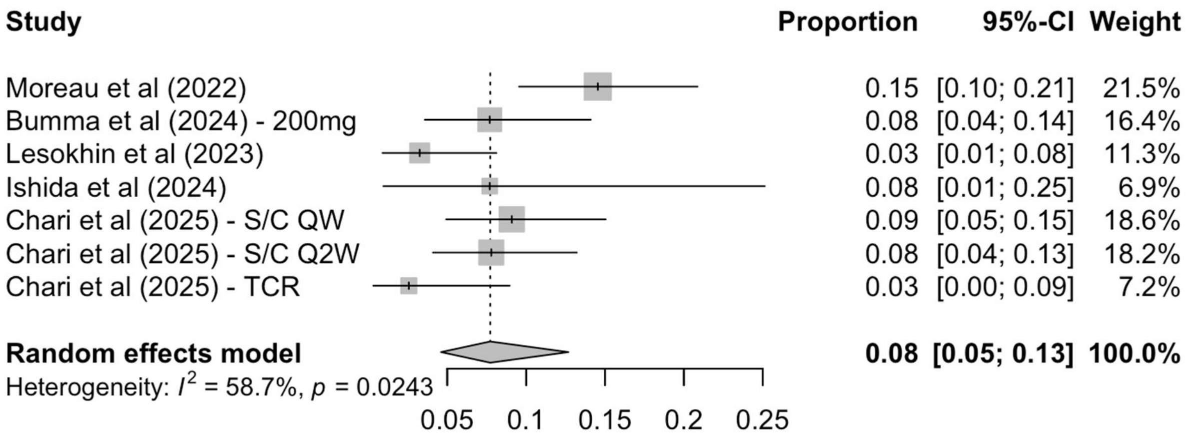

Supplementary Table S1. Risk of bias assessment per domain (adapted ROBINS-I V2)

	Confounding	Classification of interventions	Selection of participants	Deviations form intended interventions	Missing data	Measurement of outcome	Selection of reported result
Moreau et al (2022)	Moderate risk	Low risk	Low risk	Low risk	Low risk	Low risk	Low risk
Cohen et al (2025)	Moderate risk	Low risk	Low risk	Low risk	Low risk	Low risk	Low risk
Bumma et al (2024)	Moderate risk	Low risk	Low risk	Low risk	Low risk	Low risk	Low risk
Lesokhin et al (2023)	Moderate risk	Low risk	Low risk	Low risk	Low risk	Low risk	Low risk
Chari et al (2025)	Moderate risk	Low risk	Low risk	Low risk	Low risk	Low risk	Low risk
Ishida et al (2024)	Moderate risk	Low risk	Low risk	Low risk	Moderate risk	Low risk	Low risk

Supplementary Table S2. Certainty of evidence assessment by GRADE

Outcome	No. of studies	No. of participants	Pooled estimate (95% CI)	Certainty of evidence	Reasons for rating
ORR	6	850	69% (63-74%)	Low	Uncontrolled, open label trials
≥CR	6	850	42% (35-50%)	Low	Uncontrolled, open label trials, Moderate heterogeneity (I^2=60.2%, p=0.0139)
1-year DOR	6	850	71% (55-84%)	Very low	Uncontrolled, open label trials, High heterogeneity (I^2=85.1%, p<0.0001)
1-year PFS	6	850	56% (43-69%)	Very low	Uncontrolled, open label trials, High heterogeneity (I^2=86.4%, p<0.0001)
1-year OS	6	850	72% (66-77%)	Low	Uncontrolled, open label trials Moderate heterogeneity (I^2=58.3%, P=0.0254)
MRD negativity	4	431	24% (14-39%)	Very low	Uncontrolled, open label trials

					Moderate heterogeneity (I^2=58.9%, P=0.0627)<br>Missing data – MRD testing in selected subpopulations
Grade 3-4 Anemia	5	806	32% (27-36%)	Low	Uncontrolled, open label trials
Grade 3-4 Leukopenia	2	540	11% (5-20%)	Very low	Uncontrolled, open label trials<br>Moderate heterogeneity (I^2=60.8%, P=0.0537)<br>Only two studies
Grade 3-4 Lymphopenia	5	806	24% (17-32%)	Very low	Uncontrolled, open label trials<br>Moderate heterogeneity (I^2=69.6%, P=0.0031)
Grade 3-4 Neutropenia	5	806	46% (29-63%)	Very low	Uncontrolled, open label trials<br>High heterogeneity (I^2=91.9%, P<0.0001)
Grade 3-4 Thrombocytopenia	4	780	21% (17-25%)	Low	Uncontrolled, open label trials
Grade 3-4 Infections	5	806	29% (20-40%)	Very low	Uncontrolled, open label trials<br>High heterogeneity (I^2=85.5%, P<0.0001)
Any grade CRS	5	806	69% (57-79%)	Very low	Uncontrolled, open label trials<br>High heterogeneity (I^2=86.6%, P<0.0001)
Any grade Neurotoxicity	5	806	8% (5-13%)	Low	Uncontrolled, open label trials<br>Moderate heterogeneity (I^2=58.7%, P=0.0243)

Supplementary Table S3. PRISMA 2020 checklist

Section and Topic	Item #	Checklist item	Location where item is reported
**TITLE**			
Title	1	Identify the report as a systematic review.	Page 1
**ABSTRACT**			
Abstract	2	See the PRISMA 2020 for Abstracts checklist.	Pages 1, 2

Section and Topic	Item #	Checklist item	Location where item is reported
**INTRODUCTION**			
Rationale	3	Describe the rationale for the review in the context of existing knowledge.	Page 3
Objectives	4	Provide an explicit statement of the objective(s) or question(s) the review addresses.	Page 3
**METHODS**			
Eligibility criteria	5	Specify the inclusion and exclusion criteria for the review and how studies were grouped for the syntheses.	Page 4
Information sources	6	Specify all databases, registers, websites, organisations, reference lists and other sources searched or consulted to identify studies. Specify the date when each source was last searched or consulted.	Page 4
Search strategy	7	Present the full search strategies for all databases, registers and websites, including any filters and limits used.	Supplementary Material
Selection process	8	Specify the methods used to decide whether a study met the inclusion criteria of the review, including how many reviewers screened each record and each report retrieved, whether they worked independently, and if applicable, details of automation tools used in the process.	Page 4
Data collection process	9	Specify the methods used to collect data from reports, including how many reviewers collected data from each report, whether they worked independently, any processes for obtaining or confirming data from study investigators, and if applicable, details of automation tools used in the process.	Page 4
Data items	10a	List and define all outcomes for which data were sought. Specify whether all results that were compatible with each outcome domain in each study were sought (e.g. for all measures, time points, analyses), and if not, the methods used to decide which results to collect.	Page 4
	10b	List and define all other variables for which data were sought (e.g. participant and intervention characteristics, funding sources). Describe any assumptions made about any missing or unclear information.	Page 4
Study risk of bias assessment	11	Specify the methods used to assess risk of bias in the included studies, including details of the tool(s) used, how many reviewers assessed each study and whether they worked independently, and if applicable, details of automation tools used in the process.	Page 5
Effect measures	12	Specify for each outcome the effect measure(s) (e.g. risk ratio, mean difference) used in the synthesis or presentation of results.	Page 4
Synthesis methods	13a	Describe the processes used to decide which studies were eligible for each synthesis (e.g. tabulating the study intervention characteristics and comparing against the planned groups for each synthesis (item #5)).	Page 4
	13b	Describe any methods required to prepare the data for presentation or synthesis, such as handling of missing summary statistics, or data conversions.	Page 4
	13c	Describe any methods used to tabulate or visually display results of individual studies and syntheses.	Page 4
	13d	Describe any methods used to synthesize results and provide a rationale for the choice(s). If meta-analysis was performed, describe the model(s), method(s) to identify the presence and extent of statistical heterogeneity, and software package(s) used.	Page 4
	13e	Describe any methods used to explore possible causes of heterogeneity among study results (e.g. subgroup analysis, meta-regression).	Page 4
	13f	Describe any sensitivity analyses conducted to assess robustness of the synthesized results.	Page 4
Reporting bias assessment	14	Describe any methods used to assess risk of bias due to missing results in a synthesis (arising from reporting biases).	Page 5

Section and Topic	Item #	Checklist item	Location where item is reported
Certainty assessment	15	Describe any methods used to assess certainty (or confidence) in the body of evidence for an outcome.	Page 5
**RESULTS**			
Study selection	16a	Describe the results of the search and selection process, from the number of records identified in the search to the number of studies included in the review, ideally using a flow diagram.	Page 5
	16b	Cite studies that might appear to meet the inclusion criteria, but which were excluded, and explain why they were excluded.	NE
Study characteristics	17	Cite each included study and present its characteristics.	Pages 5, 6
Risk of bias in studies	18	Present assessments of risk of bias for each included study.	Page 10, Supplementary Material
Results of individual studies	19	For all outcomes, present, for each study: (a) summary statistics for each group (where appropriate) and (b) an effect estimate and its precision (e.g. confidence/credible interval), ideally using structured tables or plots.	Pages 7-10
Results of syntheses	20a	For each synthesis, briefly summarise the characteristics and risk of bias among contributing studies.	Page 5, Page 10, Supplementary Material
	20b	Present results of all statistical syntheses conducted. If meta-analysis was done, present for each the summary estimate and its precision (e.g. confidence/credible interval) and measures of statistical heterogeneity. If comparing groups, describe the direction of the effect.	Pages 7-10
	20c	Present results of all investigations of possible causes of heterogeneity among study results.	NE (small sample size)
	20d	Present results of all sensitivity analyses conducted to assess the robustness of the synthesized results.	NE (small sample size)
Reporting biases	21	Present assessments of risk of bias due to missing results (arising from reporting biases) for each synthesis assessed.	Supplementary Material
Certainty of evidence	22	Present assessments of certainty (or confidence) in the body of evidence for each outcome assessed.	Page 10, Supplementary Material
**DISCUSSION**			
Discussion	23a	Provide a general interpretation of the results in the context of other evidence.	Pages 10-12
	23b	Discuss any limitations of the evidence included in the review.	Page 12
	23c	Discuss any limitations of the review processes used.	Page 12

Section and Topic	Item #	Checklist item	Location where item is reported
	23d	Discuss implications of the results for practice, policy, and future research.	Page 12
**OTHER INFORMATION**			
Registration and protocol	24a	Provide registration information for the review, including register name and registration number, or state that the review was not registered.	Page 3
	24b	Indicate where the review protocol can be accessed, or state that a protocol was not prepared.	Page 3
	24c	Describe and explain any amendments to information provided at registration or in the protocol.	NE
Support	25	Describe sources of financial or non-financial support for the review, and the role of the funders or sponsors in the review.	Page 13
Competing interests	26	Declare any competing interests of review authors.	Page 13
Availability of data, code and other materials	27	Report which of the following are publicly available and where they can be found: template data collection forms; data extracted from included studies; data used for all analyses; analytic code; any other materials used in the review.	Page 13

Search strategy

Population: Adult RRMM patients
Intervention: Treatment with bispecific antibodies
Comparator: No control/comparator group is necessary
Outcomes: Efficacy (response rates, duration of response, survival outcomes), Safety (hematological toxicities, infections, CRS)
Study type: Clinical Trials

PubMed search algorithm
((((((Bispecific antibod*[Title/Abstract]) OR (b-cell maturation antigen[Title/Abstract])) OR (Teclistamab[Title/Abstract])) OR (Elranatamab[Title/Abstract])) OR (Talquetamab[Title/Abstract])) OR (Linvoseltamab[Title/Abstract])) OR (Cevostamab[Title/Abstract])AND((((Multiple myeloma[Title/Abstract]) OR (Relapsed multiple myeloma[Title/Abstract])) OR (Refractory multiple myeloma[Title/Abstract])) OR (Plasma cell dyscrasia[Title/Abstract])) OR (Kahler disease[Title/Abstract])AND
(((Antigen*[Title/Abstract]) OR (BCMA[Title/Abstract])) OR (FcRH5[Title/Abstract])) OR (GPRC5D[Title/Abstract])
Filters used: Clinical trials

Cochrane search string and hits
ID Search Hits
#1 multiple myeloma 6881
#2 plasma cell dyscrasia 48
#3 kahler disease 95
#4 relapsed multiple myeloma 1980
#5 refractory multiple myeloma 1879
#6 #1 OR #2 OR #3 OR #4 OR #5 6965
#7 bispecific antibod* 539
#8 teclistamab 36
#9 elranatamab 23
#10 talquetamab 20
#11 linvoseltamab 6
#12 cevostamab 2
#13 GPRC5D 9
#14 FcRH5 0
#15 #7 OR #8 OR #9 OR #10 OR #11 OR #12 OR #13 OR #14 587
#16 #6 AND #15 87A

ClinicalTrials.gov search terms
refractory multiple myeloma, bispecific antibody, other terms: kahler disease
Trial phase: 1, 2 or 3

ScienceDirect search terms
refractory multiple myeloma, bispecific antibody, clinical trial
